# Electrooculography-based continuous eye-writing recognition system for efficient assistive communication systems

**DOI:** 10.1371/journal.pone.0192684

**Published:** 2018-02-09

**Authors:** Fuming Fang, Takahiro Shinozaki

**Affiliations:** Department of Information and Communication Engineering, School of Engineering, Tokyo Institute of Technology, Yokohama, Kanagawa, Japan; Universita degli Studi di Perugia, ITALY

## Abstract

Human-computer interface systems whose input is based on eye movements can serve as a means of communication for patients with locked-in syndrome. Eye-writing is one such system; users can input characters by moving their eyes to follow the lines of the strokes corresponding to characters. Although this input method makes it easy for patients to get started because of their familiarity with handwriting, existing eye-writing systems suffer from slow input rates because they require a pause between input characters to simplify the automatic recognition process. In this paper, we propose a continuous eye-writing recognition system that achieves a rapid input rate because it accepts characters eye-written continuously, with no pauses. For recognition purposes, the proposed system first detects eye movements using electrooculography (EOG), and then a hidden Markov model (HMM) is applied to model the EOG signals and recognize the eye-written characters. Additionally, this paper investigates an EOG adaptation that uses a deep neural network (DNN)-based HMM. Experiments with six participants showed an average input speed of 27.9 character/min using Japanese Katakana as the input target characters. A Katakana character-recognition error rate of only 5.0% was achieved using 13.8 minutes of adaptation data.

## Introduction

Eye movement-based communication is extremely important for people such as patients with amyotrophic lateral sclerosis (ALS) who have lost nearly all their ability to control voluntary movements, including loss of speech and handwriting but not eye movement [1, 2]. For these patients, the most common means of communication is to have a caregiver face the patient through a transparent character board and then identify which character the patient is looking at [3]. Instead of using a caregiver, this study investigates human-computer interface systems whose input is based on eye movements. Based on whether these systems require a computer screen, they can be split into two groups. Among those that use a screen, Kate et al. [4] and Majaranta et al. [5] designed an on-screen keyboard selection system in which a user could select a key by either stopping a moving cursor using a triggering eye movement or by gazing continuously at a particular key for a fixed duration (this duration is termed “dwell time” [6]). Urbina and Huckauf developed a two-level hierarchical menu selection method [7], in which the user could select a character group from the bottom menu and then select a character from a corresponding pop-up menu by simply glancing through the relevant menu items. Ward and MacKay proposed a continuous selection method named Dasher [8, 9], in which a language model was used to predict the next likely characters. The predicted characters were displayed near the current character and then the user could simply glance from character to character without stopping for selection. The average text entry rate of the on-screen keyboard system was slow---approximately 7.0 words/min [10]---but faster rates of 13.0 and 17.3 words/min were obtained for the hierarchical selection and Dasher, respectively, because those systems did not require any dwell time. This suggests that rapid input rate is a primary concern for these a ssistive communication systems and that it can be achieved without dwell time.

For systems that do not involve selecting characters from a screen, the characters are instead represented by eye movements; a user inputs intended characters directly by performing the corresponding eye movements. Because this type of system does not require a screen, it can be implemented in a small device, which gives this approach the advantage of portability. Tsai et al. developed such a system termed “eye-writing” [11], in which the user could input a character by moving the eyes along the pre-defined path of a corresponding character stroke. Another reason this input procedure is called “eye-writing” is that it utilizes the user’s original handwriting knowledge; therefore, it is quite easy for patients to get started. In this system, biomedical signal electrooculography (EOG) [12] was used to detect eye movements and artificial neural network (ANN) [13] was applied for character recognition. To make automatic character recognition easier, the eye-written characters were separated by a pause. The numbers of changed eye movement directions between pauses were used as input for the ANN. Their study showed a recognition error rate of 27.9% for 10 Arabic numerals and four arithmetic operators input but the input rate was not reported. Lee et al. proposed a similar eye-writing system [14], in which a designated writing time of eight seconds per character was set and the user paused his/her gaze at the last point of the character stroke before the end of the designated time for character separation. In this system, the recognition was based on dynamic time warping (DTW) [15]. They reported a recognition error rate of 12.6% and an input rate of 7.5 characters/min using 26 alphabetic characters and three functional symbols (space, backspace, and enter) as the input targets. Obviously, slow input rates are a problem for these systems because they require a waiting period (which can be considered as the dwell time) between input characters. We refer to systems that require character separation and recognize each character in isolation as “isolated input systems.”

One way to improve the input rate is to continuously input characters without requiring the dwell time. However, in continuous input, the eye motions are fused; consequently, the boundaries between the motions are unclear and accurate recognition is difficult. We previously studied how to accurately recognize continuously input eye motions based on EOG for our assistive communication system [16]. In this system, five types of eye motion (“up”, “down”, “left”, “right”, and “center”) were combined to represent characters, and this representation was defined as a code protocol. Hidden Markov model (HMM) [17]-based speech recognition techniques were then applied to recognize and convert the eye motions into characters. To better handle the fused eye motions, we developed a context-dependent EOG model. When we combined this model with an N-gram language model [18], we achieved a character-recognition error rate of 0.9% for Japanese Katakana character input. Although this system has a very high input accuracy, it has a problem in that there is no intuitive connection between a motion sequence and the corresponding character; therefore, it is very difficult for users to learn the protocol.

In this paper, we propose an EOG-based continuous eye-writing recognition system. Compared to the isolated eye-writing systems, the proposed system can continuously accept characters; therefore, a faster input rate is anticipated. Compared to our previously developed code-protocol-based eye input assistive communication system described above, this proposed system is more user-friendly because it utilizes the user’s original handwriting knowledge. The recognition scheme of the proposed system is extended from the previous system. We use the same idea as the previous system to recognize the eye-written characters; i.e., we encoded eye-written characters into a sequence consisting of a set of basic eye motions and represented the coding using an input protocol. Then, we modeled these motions using a context-dependent model. Because recognizing continuous eye motions in eye-writing is more difficult than the methods used in the previous system, we then improved the recognition system by introducing a deep neural network (DNN) [13]. To address the user dependency, EOG adaptation based on DNN is also investigated. Experiments demonstrated that the proposed system can achieve a rapid input rate and a low error rate using 70 Japanese Katakana characters as input targets.

## Materials and methods

### Electrooculography (EOG)

**Fig 1:**
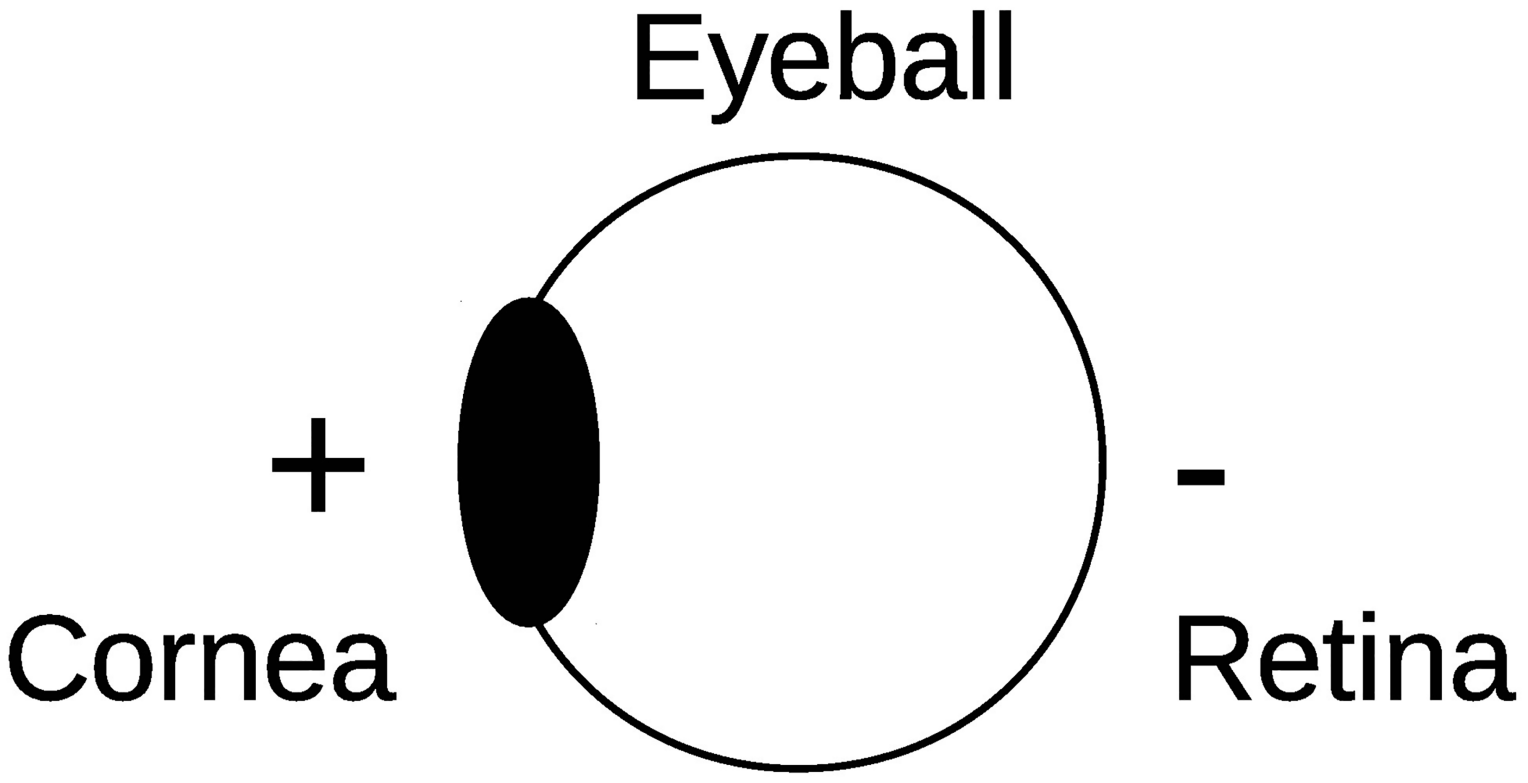
Corneoretinal potential (CRP).

An electrical potential exists between the cornea and retina of the human eye, called the corneoretinal potential (CRP), as shown in Fig 1. The corneal side has a positive charge, and the retinal side has a negative charge. The CRP can be observed as an EOG signal by attaching a set of electrodes on the skin around the eyes. Because the EOG signal reflects the eye position, it can be used to detect eye movements. The EOG signal typically shows signal amplitudes ranging between 250 and 1,000 *μ*V at a frequency of approximately 0 to 30 Hz [12]. The EOG amplitude range is larger than that of an electroencephalogram (EEG) [19], and EOG signals are easier to detect than EEG signals. An EOG can be detected even when the eyes are closed.

**Fig 2:**
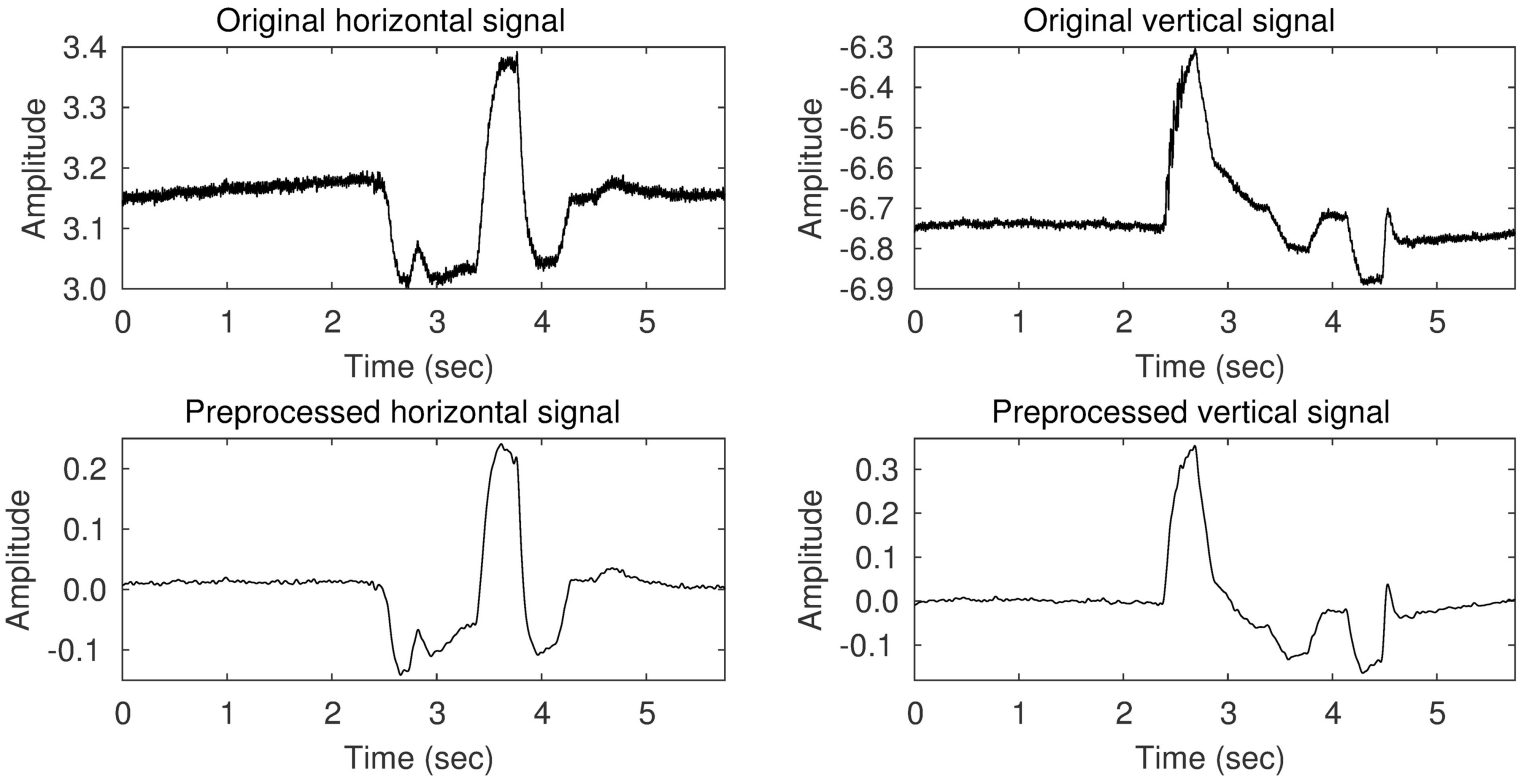
An example of a raw EOG signal and the corresponding processed signal. The signal corresponds to an eye movement sequence of “up, down, right, left, right, and down.” The top two graphs are the original signals and represent the horizontal and vertical movements, respectively. The bottom two graphs show the pre-processed signals (after applying a DC blocker and a low pass filter), which have smaller noise components.

Fig 2 shows examples of EOG signals obtained by attaching four electrodes at the left, right, top, and bottom side of the left eye. The horizontal signal was obtained by calculating the signal difference between the electrodes on the left and right sides, and the vertical signal was obtained by calculating the difference between the electrodes on the top and bottom sides. The top two graphs correspond to raw observations with no pre-processing. The main noise sources for EOG signals are electromagnetic noise from the powerline, internal noise from the measurement device, the quality of the contact between the skin and the electrodes, and other physiological sources such as electromyographic (EMG) [20] signals [21]. Among these, powerline noise can be relatively easily removed by applying a low-pass filter with a cut-off frequency below 50 Hz because the powerline frequency is generally either 50 or 60 Hz regardless of country or region. Baseline drifts due to contact quality, etc. can be removed by cutting a direct current (DC) component.

In this paper, we apply a finite impulse response (FIR) low pass filter with a cutoff frequency of 20 Hz, which is high enough to capture most of the intentional eye movements but low enough to remove undesirable high-frequency noise. To remove baseline drift, a DC blocker [22] is used whose transfer function is defined as shown in Eq 1, where *R* = 0.999. The bottom two graphs in Fig 2 correspond to the top two graphs after applying these pre-processing steps.

$$\begin{array}{c} H\left(z\right)=\frac{1-z^{-1}}{1-Rz^{-1}} \end{array}$$ (1)

### Basic statistical models

#### Gaussian mixture model (GMM)

The Gaussian distribution, N(x|μ,Σ), is one of the most basic continuous probability distributions. It is defined by two parameters: a *d*-dimensional mean vector (*μ*) and a covariance matrix (**Σ**) as shown in Eq 2.

$$\begin{array}{c} P\left(x\right)=N(x|\mu ,\Sigma )=\frac{1}{\sqrt{{\left(2\pi \right)}^{d}\left|\Sigma \right|}}exp\left(-\frac{1}{2}{\left(x-\mu \right)}^{T}\Sigma^{-1}\left(x-\mu \right)\right). \end{array}$$ (2)

While the Gaussian distribution is useful, it can model only simple distributions with a single peak. To improve modeling flexibility, the Gaussian mixture model (GMM) is widely used. The GMM is a weighted sum of multiple Gaussian distributions, as shown in Eq 3.

$$\begin{array}{c} \begin{array}{c} P\left(x\right)=\sum_{i=1}^{M}w_{i}N\left(x|\mu_{i},\Sigma_{i}\right),\\ \sum_{i=1}^{M}w_{i}=1,\text{and}\;0\leq w_{i}\leq 1, \end{array} \end{array}$$ (3)

where *w*_*i*_ is a mixture weight, and *μ*_*i*_ and **Σ**_*i*_ are the mean and covariance of the *i*-th Gaussian component, respectively. A diagonal covariance matrix is often used to balance the model complexity and improve parameter estimation accuracy.

#### Deep neural network (DNN)

A DNN consists of multiple layers of neuron units, each of which takes multiple-inputs and provides a single-output. The input to a neuron unit can be represented by a vector. The neuron unit applies an affine transformation plus a non-linear transformation to the input, as shown in Eq 4,

$$\begin{array}{c} y=h(w^{T}\cdot x+b), \end{array}$$ (4)

where **x** is the input vector, *y* is the output, **w** and *b* are a weight matrix and a bias of the affine transformation, respectively, and *h* is the non-linear transformation function. The non-linear function *h* is called an activation function. Several choices are available for *h*, including sigmoid, rectified linear units (ReLU) [23], and softmax functions.

The design of a DNN is specified by the number of layers, the number of neuron units in each layer, and the connections between the layers. The layer at the bottom is called an input layer, the ones in intermediate positions are called hidden layers, and the top layer is called the output layer. For classification purposes, the activation function of the output layer is typically a softmax function; consequently, it represents the posteriori distribution of the classes given the input data.

### Pattern recognition methods for sequential data

#### Dynamic time warping (DTW)

DTW is a method to measure the dissimilarity between two sequences of different lengths. It searches for an optimal alignment between the two sequences that minimizes a matching cost and outputs the minimum cost as the dissimilarity measure of the two sequences. For example, suppose **X** = {**x**_1_, **x**_2_, ⋯, **x**_*M*_} and **Y** = {**y**_1_, **y**_2_, ⋯, **y**_*N*_} are two vector sequences whose lengths are *M* and *N*, respectively. An alignment is a sequence of pairs of the sequence indices {(*s*_1_, *t*_1_), (*s*_2_, *t*_2_), ⋯, (*s*_*i*_, *t*_*i*_), ⋯, (*s*_*L*_, *t*_*L*_)}, where *s*_1_ = *t*_1_ = 1, *s*_*L*_ = *M*, *t*_*L*_ = *N*, max{*M*, *N*} ≤ *L* < *M* + *N*, and (*s*_*i* + 1_, *t*_*i* + 1_) is either (*s*_*i*_ + 1, *t*_*i*_), (*s*_*i*_, *t*_*i*_ + 1), or (*s*_*i*_ + 1, *t*_*i*_ + 1). Given an alignment, the matching cost is given by Eq 5.

$$\begin{array}{c} \sum_{i=1}^{L}dis(x_{s_{i}},y_{t_{i}}) \end{array}$$ (5)

where *dis*(**x**, **y**) is the Euclidean distance between two vectors **x** and **y**. While the computational cost of a direct enumeration of all possible alignments grows exponentially with the sequence length, the DTW computes the minimum distance efficiently using dynamic programming [15].

#### Hidden Markov model (HMM)

The HMM models a probability distribution over a data sequence. An HMM consists of a set of *N* states {1, 2, ⋯, *N*} each of which is associated with an output distribution and a set of directed edges assigned with state transition probabilities. It models the joint probability of a data sequence **X** = {**x**_1_, ⋯, **x**_*L*_} and an HMM state sequence **S** = {*s*_1_, ⋯, *s*_*L*_} of length *L* where *s*_*i*_ ∈ {1, 2, ⋯, *N*}, as shown in Eq 6,

$$\begin{array}{c} P\left(S,X\right)=P\left(s_{1}\right)P\left(x_{1}|s_{1}\right)\prod_{i=2}^{L}P\left(s_{i}|s_{i-1}\right)P\left(x_{i}|s_{i}\right), \end{array}$$ (6)

where *P*(*s*_1_) is an initial state probability. When an HMM is applied to an observed data sequence, **X**, the corresponding HMM state sequence **S** is usually unknown. The most likely state sequence S* is obtained by maximizing the joint probability *P*(**S**, **X**) over **S**. While direct enumeration of all possible values for **S** requires exponential computational cost for the sequence length, it can be efficiently maximized using the Viterbi algorithm [24]. Before using the HMM for pattern recognition, the output and transition probability distributions must be estimated. These estimations are based on maximizing the likelihood of training data and performed using the Baum-Welch algorithm [25].

HMM has been intensively used for speech recognition. In this field, GMM had been widely used as the output distribution; however, DNN has recently become more popular than GMM for the HMM output distribution because of its superior recognition performance. In this paper, the HMM that uses the GMM as the output distribution is referred to as GMM-HMM, and the one that uses DNN is termed DNN-HMM.

#### N-gram language model

An N-gram is a probability model for a discrete symbol sequence such as a character or word sequence. Suppose **W** = {*w*_1_, *w*_2_, ⋯, *w*_*L*_} is a symbol sequence with length *L*. By recursively applying Bayes’ theorem, the probability of **W** is decomposed to a product of the conditional probabilities of each symbol in the sequence, as shown in Eq 7. In N-gram modeling, symbol history older than *N* − 1 steps is ignored when calculating the conditional probability of the current symbol *P*(*w*_*i*_|*w*_1_, *w*_2_, ⋯, *w*_*i* − 1_), as shown in Eq 8,

$$\begin{array}{ccc} P(W) & = & P(w_{1})\prod_{i=2}^{L}P(w_{i}|w_{1},w_{2},\cdots ,w_{i-1})\\ & \approx & P\left(w_{1}\right)P\left(w_{2}|w_{1}\right)\cdots P\left(w_{N-1}|w_{1},w_{2},\cdots ,w_{N-2}\right) \end{array}$$ (7)

$$\begin{array}{c} \cdot \prod_{i=N}^{L}P(w_{i}|w_{i-N+1},w_{i-N+2},\cdots ,w_{i-1}), \end{array}$$ (8)

where *N* is the N-gram order. When *N* = 1, the history is not considered at all, and the calculation of *P*(**W**) is reduced to a simple product of the symbol probabilities, $\prod_{i=1}^{L}P(w_{i})$. In general, the larger the N-gram order, *N*, the longer the symbol history is remembered and the more flexible the model becomes. However, the number of model parameters grows exponentially as *N* increases. To balance model flexibility against parameter estimation accuracy, relatively small *N* values---approximately 1 to 5---are typically chosen based on the task.

### The previously developed code-protocol-based eye input system

**Fig 3:**
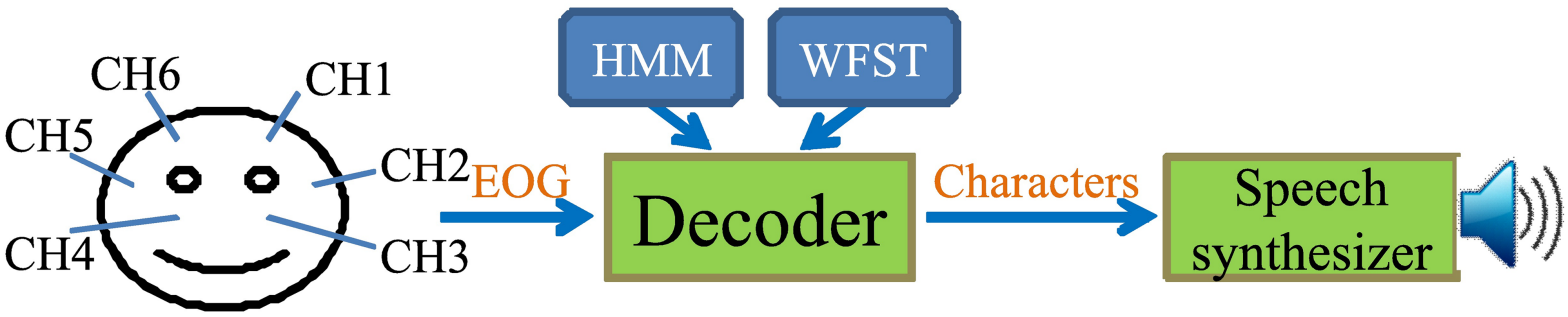
Overview of the previously developed code-protocol-based eye input system.

Fig 3 shows an overview of our previously developed code-protocol-based eye input system. This system first detected the user’s EOG signals via six electrodes attached to the skin around the eyes. Then it recognized a sequence of codes representing characters from the EOG signal using an HMM-based recognition decoder. Finally, it synthesized and output speech sound that corresponded to the recognized characters using a text-to-speech (TTS) synthesizer [26] and a loudspeaker. Using this system, the users could combine the five motion types to continuously input characters in accordance with a pre-defined code protocol. For Japanese input, each of 48 basic Japanese Katakana characters was represented by a combination of four motions, and another 22 derived Katakana characters were represented by combining a basic Katakana character and one of two diacritical marks (also represented by four motions). To represent the protocol, a weighted finite-state transducer (WFST) [27] was used, which can provide a mapping between eye motions and characters.

To effectively model continuous eye motions while considering the influence of adjacent motions, a context-dependent model was used. More precisely, this system utilized a tri-eye motion model to model the five types of eye motions, which allowed the influences of the preceding and succeeding eye motions to be considered. Because the tri-eye motion model combined three eye motions, it contained a large number of parameters and required a large amount of training data. To train a robust model with limited available data, a decision-tree-based state clustering [28] approach was used in which similar parameters were clustered into a group, and their training data were shared.

### Eye-writing input protocol

#### Basic input protocol design

**Fig 4:**
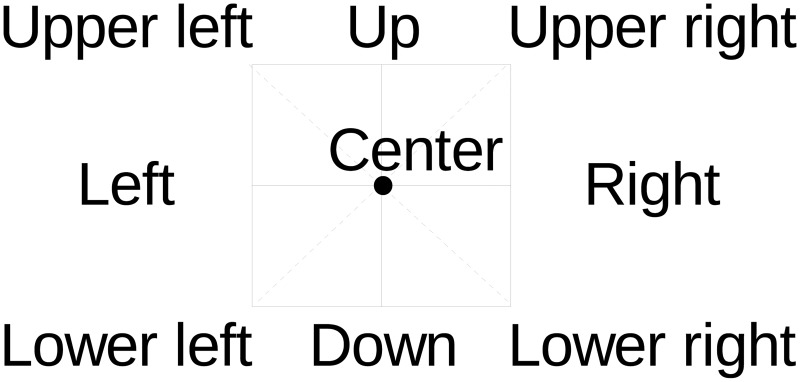
Nine of the 11 basic eye motions used for model units.

**Fig 5:**
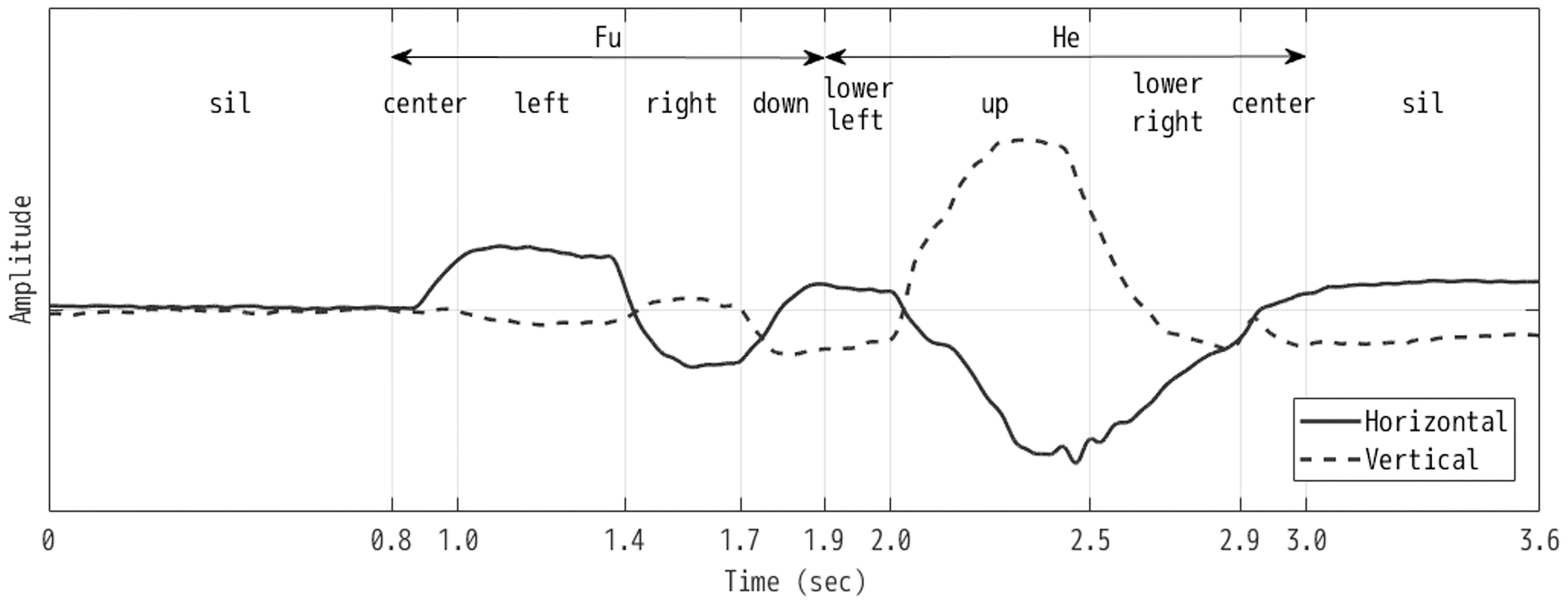
An example of alignment of the eye motion models to an EOG input. The input EOG feature is a two-dimensional vector representing horizontal (left-right) and vertical (up-down) eye movements. Two Japanese Katakana characters “fu” and “he” are continuously eye-written. The “sil” label indicates no motion is performed in the segment. The boundaries are automatically decided by the Viterbi decoding so as to maximize the posterior probability of the alignment.

When designing an eye-writing input protocol, one problem that arises is how to model the eye movements that represent characters. The most straightforward method is to model each eye gesture that corresponds to a character using a separate HMM. This approach is highly flexible in capturing character-dependent detailed motions; however, the number of parameters is large, and parameter estimation becomes difficult when data are limited. Another approach is to decompose the eye gestures into smaller basic units shared between the characters. This reduces the modeling flexibility somewhat, but the model set has a much smaller number of parameters. Consequently, an accurate model can be estimated from a limited amount of training data. This situation is analogous to the word- and phone-modeling approaches in speech recognition, where the phone-modeling approach is widely adopted (except for small-vocabulary isolated-word recognition tasks). We adopted the approach of decomposing the continuous eye gestures for recognition based on some preliminary experiments. Additionally, similar to the system described in Lee et al. [14], we introduce a lightweight constraint in eye-writing characters to assist the recognition systems in recognizing the shape: that is, the nine eye motions shown in Fig 4 and a blinking motion are defined as basic eye motions, and characters are written by combining these motions. For example, the letter “A” is eye-written by combining the eye motions of “lower left, up, lower right, left, and right.” In addition to these 10 unit eye motion models, we use “sil” model to indicate that the eye is at the neutral position without a motion. Therefore, the total number of the basic units of our system is 11. For the model training and decoding, the sil model is treated just the same way as other eye motion models. Fig 5 shows an example how these models align to input EOG signal.

#### The input protocol for Japanese Katakana characters

**Fig 6:**
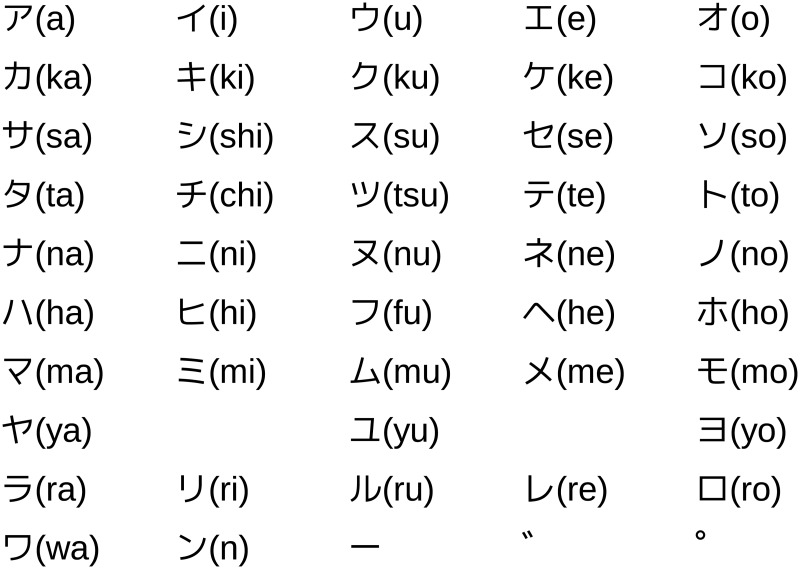
Japanese Katakana characters and their corresponding syllables.

**Fig 7:**
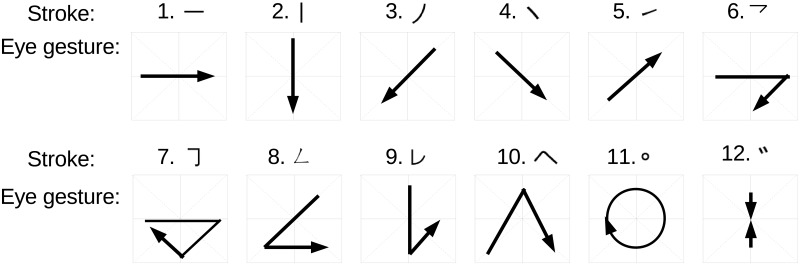
Basic Katakana strokes and the corresponding eye gestures. Stroke No. 12 is represented by a blinking motion.

Our implemented system is designed to input Japanese Katakana characters, which represent all Japanese syllables. Fig 6 shows the basic Katakana characters and their alphabet-based representations. Because Katakana characters are complex, the characters are decomposed into strokes for the eye-writing. Instead of directly eye-writing the entire character, the strokes are eye-written individually based on exaggerated eye movements corresponding to the 11 basic motions. The Katakana strokes used in this study are shown in Fig 7. With these strokes, for example, /hi/ can be input by eye-writing stroke No. 3 and 9. As an example of the stroke representation, stroke No. 11 is approximately expressed by “left, up, right, down, left”. With this eye-writing input protocol, there is a possibility that multiple characters correspond to the same input. The same problem was existed in our previous code-protocol-based system [29]. As before, we use N-gram language model to reduce the ambiguity utilizing the history of the input characters.

### HMM-based modeling of a continuous eye-writing EOG signal

The modeling method used in the proposed system is the same as the previous code-protocol-based eye input system; that is, the basic eye motions are modeled using the tri-eye motion model, and the decision-tree-based state clustering method is used to cluster similar parameters. As the output distribution of an HMM state, the GMM was used in our previous code-protocol-based system. In this study, we instead use a DNN as the output distribution in addition to GMM. The DNN-HMM model has been reported to achieve performances superior to GMM-HMM in the latest speech recognition systems [30], and it is expected that DNN-HMM will also yield improved performances in our eye motion recognition system.

#### User-dependent and user-independent modeling

HMMs are trained using a set of training data. The training data can be collected either only from a target user or collected from many different people. If the HMM is trained using data from a specific user, it is a user-dependent model. Conversely, if the HMM is trained using data from many different users, it is a user-independent model. Between these two model types, user-dependent models typically provide higher recognition accuracy because they represent only the target user’s characteristics and, therefore, tend to match well with the signal to be recognized. In contrast, while user-independent models generally provide lower accuracy, they have the advantage that any user can use the model after it is trained. Therefore, once the model has been created, new users do not need to record sample data before they can use the system.

#### User adaptation

To achieve high recognition accuracy given only a small amount of user-dependent data, applying user adaptation techniques to a user-independent model is useful. The data used for adaptation are called adaptation data. To adapt the GMM-HMM, the maximum likelihood linear regression (MLLR) [31] and maximum a posteriori (MAP) [32, 33] can be used. Both the MLLR and MAP transform mean vectors of the Gaussians to better match the adapted model to the adaptation data. To efficiently perform adaptation using a limited adaptation data, MLLR first estimates an affine transformation and then uses it to transform a set of mean vectors rather than directly estimating them, which reduces the number of free parameters that must be estimated from the adaptation data. Similarly, MAP introduces a prior distribution of the mean vectors so that the update is constrained, thus reducing the effective freedom in the parameter estimation. To adapt the DNN-HMM, we simply re-train the DNN.

### Power analysis

We did not perform the power analysis to calculate the sample size in our experimental design. None of the related researches on eye motion recognition had performed the power analysis, and the effect size was unknown. In our experiment comparing DTW and HMM, we report the effect size and power as a post hoc power analysis.

### Ethics

This research was approved by the Ethics Review Committee of Tokyo Institute of Technology (approval number: 2014083). To record EOG data, participants were recruited through a public advertisement published on this project’s web page in late July of 2016. Only healthy adult applicants were subject to recruitment. We explained the process to the applicants both verbally and in written form, including the recording method and the equipment, the uses of the recorded data (for our experiments and for building an open EOG database after removing user identification), the remuneration of 1,000 Japanese yen per hour, and the planned 10 hours of total working time for every applicant. The recording had no risk of electric shock because the recording device was completely separated from the powerline and used batteries. If injuries were to occur, insurance was available for medical treatment. The participants joined the recording of their own free will, and they had the option to quit without restriction at any time. We accepted the first six applicants, all of whom consented to participate in the recording process after the explanation. The consent of each participant was obtained in written form.

## Results and discussion

### Data recording

To evaluate the proposed system, we constructed an EOG database using data from the six healthy participants. One of the participants was female; the others were males. Their ages ranged from 21 to 31. Additionally, one participant was an eye-writing expert, but all the other participants were new to the task. The EOG recording device was a commercial biomedical amplifier, BlueGain (Cambridge Research Systems Ltd.), which was powered by a pair of double-A batteries. The recorded data was transferred to a personal computer via a Bluetooth. Four measurement electrodes were attached to the left, right, upper, and lower sides of the left eye, respectively. Additionally, one reference electrode was attached between the left and right eyebrows. The distances between the center of the eye and the left, right, upper, lower, and reference electrodes were approximately two, two, four, four, and three centimeters, respectively. The electrodes were medical Ag/AgCl surface electrodes. A two-channel (horizontal and vertical) EOG signal was obtained by taking the differences of the signals between the electrodes on the left and right sides and those between the upper and lower sides, respectively. Therefore, the EOG signal from the recording device was a sequence of two-dimensional EOG vectors. The sampling rate was 1.0 KHz.

The participants were instructed to sit on a chair during the recording. Then, two types of EOG data were recorded: isolated data and continuous data. The isolated data were recorded by eye-writing the 12 kinds of Katakana strokes in isolation in accordance with the input procedure of the isolated eye-writing system by Lee et al. [14]. These data were collected to make comparisons possible between the recognition performance of a DTW-based baseline system (our implementation of Lee et al.’s system) and our HMM-based system during the evaluation. The continuous data were recorded to evaluate the input rate and accuracy of the continuous eye-writing system. These data are available from the S1, S2 and S3 Databases. The details of the two types of datasets are described below.

#### Isolated data

In general, each of the 12 types of strokes was recorded 10 times for each participant, but a few strokes were recorded nine or 11 times. In total, 724 strokes were recorded from all the participants. For the baseline system, a set of calibration data is required to calibrate eye rotation angle due to distortions from the electrode attachment positions [14]. The calibration data were acquired from nine gaze positions where the eye rotation angles were known. In our experiment, calibration data were recorded for each participant before recording the isolated data.

#### Continuous data

The continuous data consisted of both training data and test data. The training data were recorded by asking the participants to write a selected set of 150 words repeatedly using Katakana characters. These words were selected from the Corpus of Spontaneous Japanese (CSJ) [34] and intended to maximize the number of triple eye motions contained in the words. The lengths of the words were between one and three Katakana characters (2.8 on average). In general, each word was repeatedly recorded five times, but a few words were recorded between three and eight times. To record these data, the recording period was split into two or three separate sessions for a participant and approximately 250 to 500 words were recorded during each session. All the electrodes were attached and removed for each session. In total, the recorded training data consisted of 587.2 minutes (9.8 hours) for all the participants.

The test data was recorded by eye-writing 25 sentences, which were selected from the CSJ to maximize the number of required triple eye motions. The sentences ranged in length from five to 20 Katakana characters; the average length was 15.4 characters. To record the test data, each participant eye-wrote each of the sentences one time during a single session. In total, the recorded test data consisted of 94.8 minutes (1.6 hours) for all the participants.

Because it was not possible to read the text instructions during eye-writing, the participants were asked to memorize each word or sentence before eye-writing it. To become accustomed to using the eye-writing system, all participants practiced using a subset of the words and sentences for around 5 times.

### Experimental setup

We first compare the recognition performance of the baseline DTW-based and the proposed HMM-based recognition systems in isolated recognition conditions. We did this because while the input rate of the baseline isolated input system is fixed, its recognition performance depends on the task. Then we evaluated our system in the continuous recognition condition. A two-tailed *t*-test was used to perform statistical comparisons. The setups used for eye-writing recognition are described below.

#### EOG features for recognition

As explained in the data recording section, the EOG signal is stored as a sequence of 2-dimensional vectors with the sampling frequency of 1.0 KHz. For isolated recognition conditions, the raw EOG signal was downsampled to 125 Hz after the median filtering following the procedure in [14]. For continuous recognition, the raw EOG signal was downsampled to 50 Hz after applying the DC blocker and the low pass filter. We refer to these filtered 2-dimensional EOG signals as baseform EOG features.

To improve the recognition performance, we expanded the baseform EOG features by adding delta and delta-delta features corresponding to the speed and acceleration of the change of the signal, respectively. Further, every two vectors were joined and the data rate was halved. The resulting vector has 12 (= (2 + 2 + 2) × 2) dimensions. We refer to these features as expanded EOG features. The sampling rate of the expanded EOG features for the isolated recognition was 62.5 Hz, whereas it was 25 Hz for the continuous recognition.

#### HMM training

For GMM-HMM training, two steps were conducted: parameter initialization and parameter learning. In the parameter initialization stage, the state transition probabilities were initialized to 0.5, and the output distribution of each state was initialized with a single Gaussian whose mean vector and covariance matrix were set to equal to the global mean vector and diagonal covariance matrix of the entire training dataset. In the parameter learning stage, the initialized HMMs were trained by the Baum-Welch algorithm with five iterations; then, each Gaussian was split into two to create the GMM, and the training and splitting processes were performed iteratively until the required number of Gaussian components was obtained in the GMM. Finally, the training was applied again.

The DNN-HMMs were made from the GMM-HMM, replacing GMM with DNN as the output distribution. The DNN was initialized by the Xavier initialization method [35], and a back-propagation algorithm [36] was used to train the DNN. The initial learning rate for the back-propagation was set to 0.05. The momentum, mini-batch size and number of training epochs were set to 0.9, 128, and 20, respectively. To evaluate the recognition performance of the DNN during the training iterations, 1% of the training data were randomly held out. If the recognition performance on the held-out data decreased at the end of an epoch, the learning rate was halved for the next epoch. For adaptation, the learning rate and epoch size were set to 0.01 and 15, respectively.

#### Setup for isolated eye-writing recognition

The baseform and expanded EOG features were used as the input to DTW and HMM and compared. We used 10-fold cross-validation for performance evaluation. Each stroke was represented by a template for the DTW approach and modeled by a unit HMM for the HMM approach. For the DTW, no constraint was introduced to the search scope: a full search was performed.

The unit HMM had four states and a left-to-right structure. For the output distribution of the HMM, we used a GMM with 16 Gaussian components. The Hidden Markov Model Toolkit (HTK) [37] was used to estimate the parameters of the HMM. Both the DTW template and HMM were user-dependent. Precision, recall, and F1 scores averaged over all strokes and all participants were used as recognition performance measurement criteria. The definitions of precision, recall, and F1 scores are as follows:

$$\begin{array}{c} Precision=\frac{TP}{TP+FP}, \end{array}$$ (9)

$$\begin{array}{c} Recall=\frac{TP}{TP+FN}, \end{array}$$ (10)

$$\begin{array}{c} \text{and}\;\;F1=2\cdot \frac{Precision\cdot Recall}{Precision+Recall}, \end{array}$$ (11)

where TP, FP, and FN are the number of true positives, false positives, and false negatives, respectively.

#### Setup for continuous eye-writing recognition

The basic eye motions were modeled as tri-eye motion models and the decision-tree-based state clustering method was used in the training process. All the HMMs had a left-to-right structure and contained four states. For the GMM-HMM, a Gaussian mixture distribution with 16 components was used as the state output distribution with the expanded EOG features. For the DNN-HMM, a DNN consisting of four layers was used to model the state output distribution in which the input layer had 132 units, the first and second hidden layers had 200 and 100 units, respectively, and the number of units in the output layer was equal to the number of HMM states. The activation functions of the hidden and output layer were ReLU and softmax, respectively. The input to the DNN was created from the expanded EOG feature vector by applying splicing and principal component analysis (PCA) [38]. The splicing was intended to augment the feature vector by attaching five preceding and succeeding vectors to the current vector in each time frame to better model the context effect. The PCA was applied to reduce the correlation between dimensions.

For the user-independent recognition experiments, user-independent models were trained by user based cross-validation which employed all the training data except that from the participant being tested. The tri-eye motion model initially had 592 states. These were reduced to 63.6 on average after applying the state clustering. For the user-dependent model, we randomly selected 100 words from the target participant as training data to investigate the recognition accuracy possible when only a small amount of user-specific data is available. The user-dependent model initially had 146 states, but these were reduced to 44 after the decision state clustering. Due to the smaller amount of training data compared to the user-independent model, a smaller number of states was selected. For user adaptation, a portion of the target participant’s training data was used as adaptation data.

For the language modeling, we used a character N-gram with a vocabulary size of 70 that were trained from CSJ. Unless explicitly mentioned, an N-gram order of 5 was used. The 25 sentences used as the test sentences were excluded from the training data. The recognition experiments were conducted offline using an Intel Xeon CPU running at 2.67 GHz; the real-time factor (RTF) was less than 0.85. To measure the input accuracy of the continuous eye-writing recognition system, we used the Katakana character error rate (CER) [16], in which substitution, deletion and insertion errors were considered. A substitution error means that an output differs from the corresponding input character. A deletion error means that an input character was not output. An insertion error means that no character was input but a character was output.

HTK was used for the GMM-HMM training and adaptation, while the training and adaptation of DNN was conducted using our developed tool (see the S1 Source Code). The SRILM [39] toolkit was used for N-gram training. The decoder for the continuous recognition was developed by us and the code is available from the S2 Source code. The tools, S1 and S2 Source Codes, are designed and implemented aiming to evaluate and confirm the effectiveness of the proposed continuous eye-writing method.

### Recognition performance of DTW and HMM in isolated recognition

Table 1 shows precision, recall, and F1 score of the DTW and HMM averaged over the six participants. As shown, the HMM achieved significantly better scores than the DTW-based recognition on all measures. The F1 score obtained with HMM was more than 7% above that of DTW when either the baseform or expanded EOG features were used, and the differences were statistically significant with *p* = 0.017 and 0.031, respectively, by the two-tailed paired t-test with the sample size 6. The Cohen’s *d* effect sizes were 1.43 and 1.21, the Pearson’s correlations were 0.934 and 0.928, and the powers were 0.80 and 0.66 when the significance level was 5%. The superiority of HMM is because HMM models the gestures using a probability distribution while DTW simply computes the Euclidean distance between a template and the observation. Therefore, HMM can model the degree of variance of the signals while DTW cannot.

**Table 1:** Average precision, recall, and F1 score of the DTW- and HMM-based Katakana strokes recognition systems. The scores were evaluated using the baseform and the expanded EOG features.

EOG feature	Baseform		Expanded	
Method	DTW	HMM	DTW	HMM
Precision	0.782	0.850	0.781	0.872
Recall	0.768	0.842	0.771	0.859
F1	0.775	0.846	0.776	0.865

When the baseform and the expanded EOG features are compared, the expanded features yielded similar or better performance. In particular, the expanded features always resulted in a better performance than did the baseform features when HMM was used. This result probably occurred because HMM is more robust to noise than DTW and, therefore, more able to take advantage of the delta and delta-delta features, which are susceptible to noises in the observations.

### Evaluation of the continuous eye-writing recognition system

#### Input rate

Table 2 shows the Katakana input rate of the six participants using the proposed continuous eye-writing system. On average, the participants achieved an input rate of 27.9 Katakana character/min. Compared to the baseline isolated eye-writing system where the input rate was approximately 1 character every 8 seconds or 7.5 character/min, the proposed system improved the input rate by 3.7 times. Obviously, eye-writing continuously without requiring a waiting time between characters greatly improves the input rate. In addition, although the expert (001) achieved the highest rate, there was little difference between the expert and some participants (such as participants 002 and 004). This suggests that users can quickly become proficient and reach high input rates relatively easily. Participant 005 had the slowest input rate of 19.9 character/min because this participant eye-wrote slowly and deliberately. Nevertheless, the slowest input rate of the continuous eye-writing system was 2.7 times faster than the baseline isolated eye-writing system. Additionally, the input rate varied from participant to participant, revealing an advantage of the continuous eye-writing recognition system: users can input characters at their own pace.

**Table 2:** Katakana input rate of six participants using the continuous eye-writing system.

	Participant ID						Average
	001	002	003	004	005	006	
Input speed (Katakana/min)	34.0	31.0	24.4	29.9	19.9	28.1	27.9

#### Recognition performance by user-independent and user-dependent systems

Table 3 shows the average Katakana CERs of the continuous eye-writing system. When tested with the user-independent model, both GMM-HMM and DNN-HMM yielded large error rates. However, when tested with the user-dependent model, the Katakana CERs were greatly reduced and high recognition performance was achieved. This result occurred because of the large individual differences that existed among the participants, which involved both EOG signal-level differences and the shapes the participants used in eye-writing the Katakana strokes. However, compared to the GMM-HMM, the DNN-HMM improved the recognition performance regardless of whether the user-independent or user-dependent model was used, showing that DNN-HMM is more accurate than GMM-HMM for EOG-based eye motion recognition.

**Table 3:** Katakana character-recognition error rates (%) of the continuous eye-writing system.

	GMM-HMM	DNN-HMM
User-independent	19.1	18.8
User-dependent	6.5	6.2

**Fig 8:**
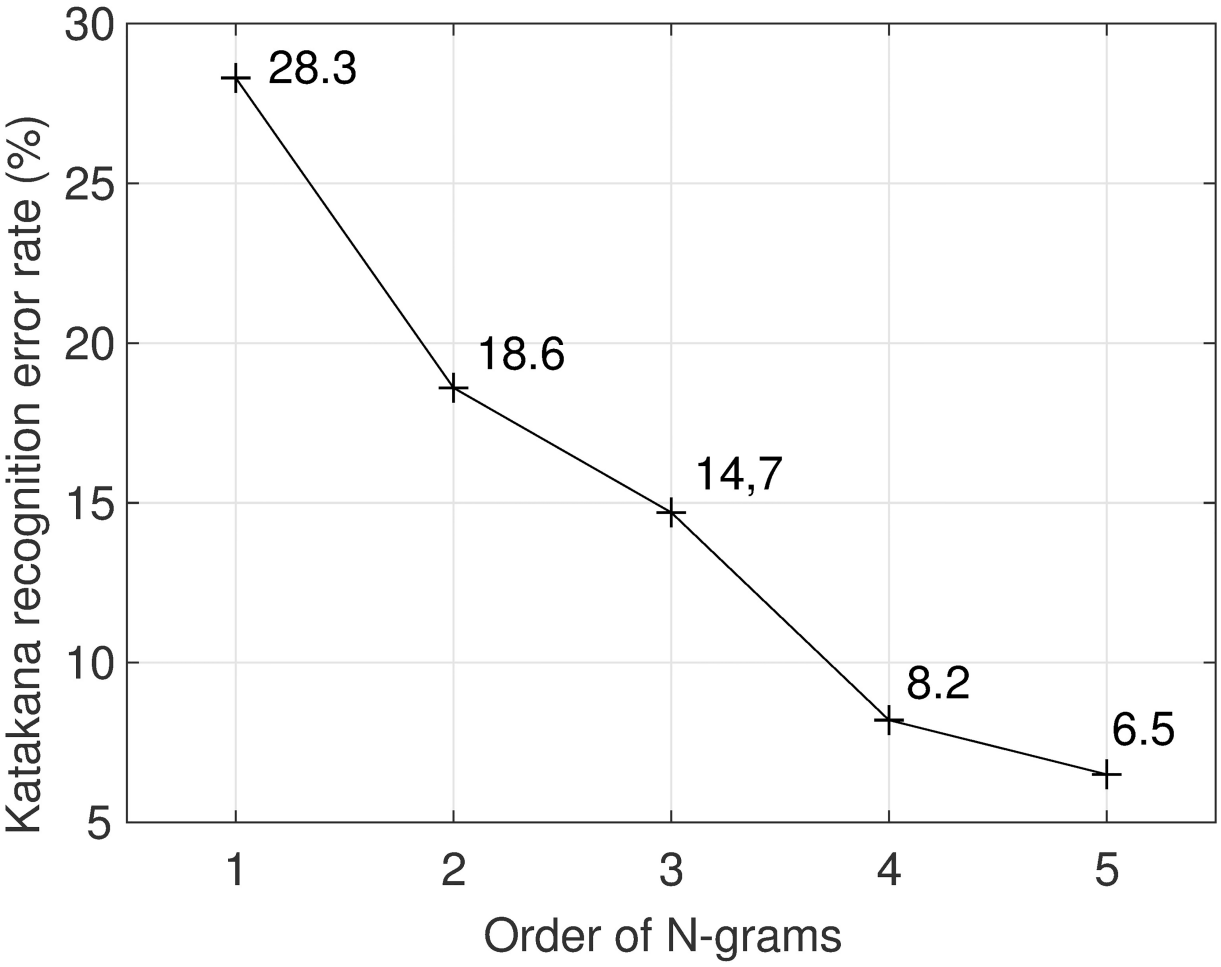
N-gram order and the Katakana recognition error rate.

To analyze the effect of using character N-gram in the recognition, Fig 8 shows the relationship between N-gram order and the Katakana CERs. The recognition is based on the user-dependent GMM-HMM. As Fig 8 shows, the error rate was reduced by increasing the N-gram order from 1 to 5. The CER when using a 1-gram was 28.3%, while it was 6.5% when a 5-gram was used.

#### User adaptation

**Fig 9:**
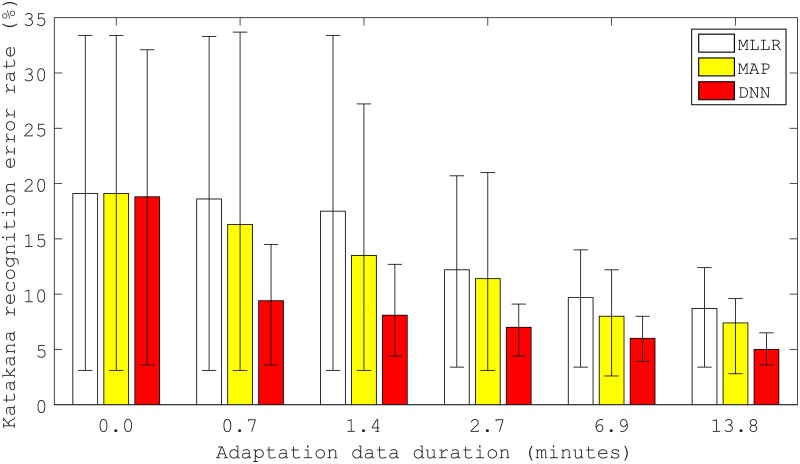
Average, maximum and minimum Katakana recognition error rate of user-independent and user-adapted models.

Fig 9 shows the average, maximum and minimum Katakana CERs of user-adapted EOG models with varying amounts of adaptation data. The horizontal axis indicates the amount of adaptation data. In this experiment, 5, 10, 20, 50, and 100 words were randomly selected from the training data of each participant to recognize. The duration of these data corresponded to 0.7, 1.4, 2.7, 6.9, and 13.8 minutes, respectively. A duration of 0.0 minutes means that the user-independent model was used. Because the MLLR and MAP used the same user-independent GMM-HMM as the initial model, their initial error rates were the same. As the figure shows, the error rate was reduced using adaptation compared to the user-independent model. Furthermore, larger amounts of adaptation data progressively reduced the error rate. Compared to GMM-HMM-based adaptation, the DNN-HMM-based adaptation achieved a smaller error rate regardless of the amount of adaptation data used. In particular, DNN-HMM achieved an error rate of only 5.0% using 13.8 minutes of adaptation data---a rate smaller than the performance of the user-dependent model trained using the same 13.8 minutes of data (see Table 3). This suggests that a user-dependent model cannot be trained to a high level of accuracy using a small amount of data, but an adapted model obtained from a well-trained user-independent model can reach a high level of accuracy with only a small amount of user-specific data.

When 13.8 minutes of adaptation data was used, the average error rate was reduced from 19.1% to 8.7% by MLLR (*p* = 0.063), from 19.1% to 7.4% by MAP (*p* = 0.046), and from 18.8% to 5.0% by DNN-HMM adaptation (*p* = 0.046). The corresponding relative error reduction rates of MLLR, MAP, and DNN-HMM adaptation were 54.5%, 61.3%, and 73.4%, respectively.

### Limitations

Although the RTF of our system is less than 1.0, there is a delay before the decoding output is obtained after the input is given. This is because some future input must be known to uniquely identify the current input: we make use of the context information using the character N-gram. The amount of delay depends on the input data, the N-gram order, and the decoding algorithm. In our decoder implementation that supports progressive output based on Viterbi beam search, the character is output after all competing recognition hypotheses having smaller posterior probability than a threshold are removed. Consequently, the delay is short when there are few competing hypotheses but can be long when many competing hypotheses exist. Because the long delay reduces the interactivity even though the input speed is fast, some mechanism to reduce the maximum delay should be introduced.

## Conclusion

In this paper, we proposed a continuous eye-writing recognition system for patients with locked-in syndrome. People using this system can input eye-written characters continuously. Because this system requires no waiting time between characters, it achieves input rates higher than conventional isolated eye-writing systems in which input characters must be separated by a pause to simplify the automatic recognition process.

The experiments in this study used Japanese Katakana as input characters. An EOG database was recorded from six healthy participants. The proposed system achieved an average input rate of 27.9 Katakana character/min---a rate 3.7 times faster than the baseline isolated eye-writing system. The proposed DNN-HMM outperformed the GMM-HMM system on all user-dependent, user-independent and user-adapted models. The DNN-HMM achieved the best Katakana character-recognition error rate of 5.0% using 13.8 minutes of adaptation data.

Our future work includes further improving the input rate and recognition performance of eye-writing systems. One approach for improving the recognition performance might be to utilize a convolutional neural network (CNN) [40]. We expect that a CNN can capture the variance in eye-written characters and reduce the amount of adaptation data required. A long short-term memory (LSTM) [41]-based language model could also be considered. To reduce the maximum delay, the search algorithm should be improved by introducing an active mechanism to control the output delay. Finally, the system should also be evaluated using EOG signals from ALS patients.

## Supporting information

S1 Database Isolated data. All the original isolated EOG data used in the experiments are contained in this database.
(ZIP)

S2 Database Continuous dataset 1. All the original continuous EOG data of participant 001, 002, and 003 used in the experiments are contained in this database.
(ZIP)

S3 Database Continuous dataset 2. All the original continuous EOG data of participant 004, 005, and 006 used in the experiments are contained in this database.
(ZIP)

S1 Source Code DNN training toolkit. This repository contains all the source code of the DNN training toolkit used in this paper.
(ZIP)

S2 Source Code Decoder. This repository contains all the source code of the decoder used in this paper.
(ZIP)
